# The Functional Components and Hepatic Protective Mechanism of Wolfberry Vinegar by Mixed-Culture Fermentation

**DOI:** 10.3390/foods14071278

**Published:** 2025-04-07

**Authors:** Xiao Qiang, Man Zhao, Ting Xia, Qi Wang, Junwei Yu, Yunru Song, Huimin Zhang, Changsheng Qiao, Min Wang

**Affiliations:** 1 State Key Laboratory of Food Nutrition and Safety, Key Laboratory of Industrial Fermentation Microbiology, College of Biotechnology, Tianjin University of Science and Technology, Tianjin 300457, China; qiangxiao0907@163.com (X.Q.);; 2 Ningxia Zhongning Goji Industrial and Innovative Research Institute Co., Ltd., Zhongwei 755000, China; 3 Qiyuantang (Ningxia) Biotechnology Co., Ltd., Zhongwei 755000, China; 4 Tianjin Huizhi Baichuan Biological Engineering Co., Ltd., Tianjin 300457, China

**Keywords:** wolfberry, vinegar, mixed-culture fermentation, alcoholic liver injury, PI3K/Akt/NF-κB

## Abstract

Wolfberry (*Lycium barbarum* L.), as a kind of combination of medicine and food, is rich in antioxidant components. However, the deep-processed products of wolfberry need to be developed to improve its added value. This study aimed to investigate the nutrients, active antioxidant ingredients, and liver-protective mechanism of mixed-culture fermented wolfberry vinegar (MFV). The results showed that MFV had significantly higher protein and significantly lower fat content than wolfberry juice before fermentation, indicating that MFV was a healthy product. The active ingredient content, which included total phenolics, total flavonoids, polysaccharides, betaine, and antioxidant activities, was significantly increased in MFV after mixed-culture fermentation. Moreover, MFV improved histopathological changes and reduced liver biochemical indicators in alcohol-treated mice, indicating the improvement of liver function. In addition, MFV effectively alleviated alcohol-induced liver injury by increasing the expression of alcohol metabolizing enzymes and inhibiting CYP2E1 activity. MFV regulated the equilibrium between pro-oxidant and antioxidant levels by downregulating pro-oxidant markers and upregulating antioxidant markers. Furthermore, MFV reduced the levels of inflammatory indexes by inhibiting the PI3K/Akt/NF-κB signaling pathway. These results suggest that MFV is a healthy food for liver protection, which provides a strategy for deep-processed products of wolfberry.

## 1. Introduction

Wolfberry (*Lycium barbarum* L.) is a kind of traditional medicine and food belonging to the *Solanaceae* family, with health-protective applications that span over 2000 years in China [1]. The wolfberry is mainly produced in Ningxia, which is an important medicinal and edible characteristic of this agricultural product [2]. Previous research reported that the nutritional components of wolfberry include 47.2% carbohydrates, 13.9% protein, and 1.5% fat, as well as trace elements and minerals [3]. In addition, it contains active antioxidant ingredients, including polyphenols, flavonoids, carotenoids, betaine, and polysaccharides [4]. These compounds exert antioxidant activity by raising enzymatic capacity and removing oxygen free radicals [5]. Previous studies have shown that wolfberry has antioxidant, hypoglycemic, liver-protective, antitumor, and antiaging effects [6,7]. Although wolfberry has a high yield, it is difficult to preserve, with a 25% processing conversion rate [2]. Therefore, there is a need to develop wolfberry products to preserve their nutritional and health-promoting benefits.

The liver is essential for many biological functions and is involved in the metabolism of both endogenous and external toxins [8]. Alcoholic liver disease (ALD) is a common clinical disease related to alcohol abuse, which seriously endangers human health. It has been reported that 90% of long-term alcoholics have alcoholic fatty liver disease, and about 50% have inflammation and fibrosis, leading to cirrhosis and liver cancer [9]. The cause of liver injury is an excessive accumulation of reactive oxygen species, leading to lipid peroxidation in liver cells and causing structural and functional abnormalities [10]. The conventional liver protection drugs have been used in the clinic, but they induce a series of long-term adverse reactions [11]. Many studies have shown that by lowering oxidative stress, natural compounds with antioxidant activity can prevent liver damage [12].

Fermentation is an effective biotechnology for improving nutritional value and facilitating fruit preservation. Fruit vinegar is the most common fermented beverage, which is made by two successive fermentations [13]. Moreover, some nutritional and functional components in fruit vinegar come from the fruit itself; more active ingredients are produced by fermentation, such as polyphenols, flavonoids, and polysaccharides [14]. Several studies showed that fermented fruit vinegar has certain antibacterial, antioxidant, liver protection, and anti-fatigue effects [15,16]. Recent studies have reported that lactic acid bacteria are used as fermentation agents, making important contributions to improving the flavor and increasing functional value of food [17]. Our previous research found that the antioxidant abilities of MFV were higher than those of single-strain fermentation vinegar. However, the mechanistic impacts of wolfberry vinegar produced by mixed-culture fermentation on liver injury are still unclear.

In this study, wolfberry juice was used as a raw material to produce vinegar by mixed-culture fermentation using yeast and lactic acid bacteria. The nutrients, active ingredients, and antioxidant activity were detected in MFV. In addition, the liver protection mechanism of MFV was investigated in vivo. This study offers a theoretical foundation for creating deep-processed wolfberry products and the application of liver protection.

## 2. Materials and Methods

### 2.1. Raw Materials and Chemicals

Wolfberry juice (WJ) was produced by crushing the fresh wolfberry (Ningxia, China). Active dry wine yeast RW was purchased from Angel Yeast Co., Ltd. (Beijing, China). *Acetobacter pasteurianus* AC2005 was procured from Tianjin University of Science and Technology (Tianjin, China). Silymarin was purchased from Pfizer Pharmaceuticals (Beijing, China). The ALT, AST, AKP, LDH, TC, and TG detection kits were obtained from Nanjing Jiancheng Biological Product (Nanjing, China). Specific antibodies were purchased from Cell Signaling Technology, Inc. (Beverly, MA, USA).

### 2.2. Production of Mixed-Culture Fermented Vinegar of Wolfberry

A certain amount of sucrose was added to WJ (1L) to adjust the sugar content to 22 Brix, and the pH value was adjusted to 6 with citric acid. The WJ was incubated at 22 °C for 6 days with active dry wine yeast RW (0.20%), *Lactiplantibacillus plantarum* L46 (0.20%), and *Lactiplantibacillus helveticus* H1(0.20%) to obtain mixed-culture fermented wolfberry wine (MFW). Alcohol fermentation was terminated when the sugar content of the fermentation broth reached 15 Brix. *Acetobacter pasteurianus* AC2005 was activated at 30 °C for 24 h to obtain the acetic acid bacterial culture solution, and then it was added to the MFW (about 850 mL). MFW was fermented at 30 °C for 6 days in a shaker incubator (180 rpm/min). MFV (830 mL) was obtained when the acetic acid content stabilized. Fermentation was carried out in triplicate to reduce the inter-batch differences in MFV.

### 2.3. Determination of Nutritional Components

The content of carbohydrate, fat, and protein was detected by Tian et al. [2], which was conducted as described in GB/T15038-2006 [18], GB 5009.6-2016 [19], and GB 5009.5-2016 [20], respectively.

### 2.4. Determination of Active Ingredients

The total phenolic content (TPC), total flavonoid content (TFC), and levels of *Lycium barbarum* polysaccharide (LBP), carotenoids, and betaine were detected using the method of Xia et al. [21]. The TPC was detected by the Folin-Ciocalteu method. The colorimetric assay technique was used to determine the TFC. The LBP level was determined by the phenol–sulfuric acid method, and a betaine assay kit was used to assess the samples’ betaine concentration.

### 2.5. Antioxidant Activity Analysis

The DPPH, ABTS, and FRAP were measured according to the method of Xia et al. [21]. Assay kits were used to detect ABTS and FRAP.

### 2.6. Animal Experiments

All animal experiments were performed by the guidelines of the institutional animal ethics committee. Male ICR mice (5–6 weeks old, 18–22 g, Beijing Vital River Laboratory Animal Technology Co., Ltd., Tianjin, China) were raised in a pathogen-free environment provided by the Animal Ethics Committee of Nankai University (SYXK2020-0007). Four groups (n = 8) of ICR mice were placed at a constant temperature (22 ± 2 °C, 55 ± 5% humidity) and were randomly assigned to the following groups: control group (CON), model group (MOD), silymarin group (100 mg/kg b.w., SLM), and MFV group (2.5 mL/kg b.w., MFV). The control group was administered distilled water for 30 days, and the model group was orally administered alcohol every day with a weekly increase in dose (2, 4, and 6 g per kg b.w.). From the 21st to the 30th, the administration was consistently maintained at the highest dose of 6 g/kg body weight. The SLM and MFV groups were administered 100 mg/kg b.w. SLM and 2.5 mL/kg b.w. MFV for 30 days, respectively, and then with alcohol 2 h later. Animal liver and blood samples were collected on the last day.

### 2.7. Histopathological Observation

Liver tissues were fixed in 4% paraformaldehyde and embedded in paraffin (5 μm). The samples were dyed with hematoxylin–eosin (H&E). A microscope was used to view the stained slices.

### 2.8. Analysis of Biochemical Indexes

The levels of aspartate aminotransferase (AST), alanine aminotransferase (ALT), alkaline phosphatase (AKP), lactate dehydrogenase (LDH), triglycerides (TGs), and total cholesterol (TC) in serum and hepatic levels of alcohol dehydrogenase (ADH), acetaldehyde dehydrogenase (ALDH), superoxide dismutase (SOD), catalase (CAT), glutathione (GSH), glutathione peroxidase (GSH-Px), and malondialdehyde (MDA) were measured by commercial kits (Nanjing Jiancheng Biological Technology Co., Ltd., Nanjing, China). The hepatic levels of cytochrome P450 (CYP2E1), reactive oxygen species (ROS), 8-hydroxy-2′-deoxyguanosine (8-OHdG), and 4-hydroxynonenal (4-HNE) were measured by an ELISA kit (Shanghai Enzyme-linked Biotechnology Co., Ltd., Shanghai, China).

### 2.9. Measurement of Liver Inflammatory Cytokines

The levels of transforming growth factor-β1 (TGF-β1), interleukin-1β (IL-1β), interleukin-6 (IL-6), and tumor necrosis factor-α (TNF-α) in the liver were determined using ELISA kits in accordance with the manufacturer’s instructions.

### 2.10. Western Blotting Analysis

The liver tissues were electro-transferred to the polyvinylidene fluoride membrane. The membranes were sealed and incubated with the primary antibodies and secondary antibodies at 25 °C. Finally, protein expression was visualized on the membranes by an Odyssey infrared imaging system.

### 2.11. Statistical Analysis

The GraphPad Prism 8.0.1 program was used to show the data as mean ± standard deviation (S.D., n = 3). One-way analysis of variance was used to evaluate the data by SPSS 26.0 software, and the Tukey method was used for post hoc tests. *p* < 0.05 suggested significant differences.

## 3. Results and Discussion

### 3.1. Nutritional Components in Wolfberry Juice and Mixed-Culture Fermented Wolfberry Vinegar

**Figure 1:**
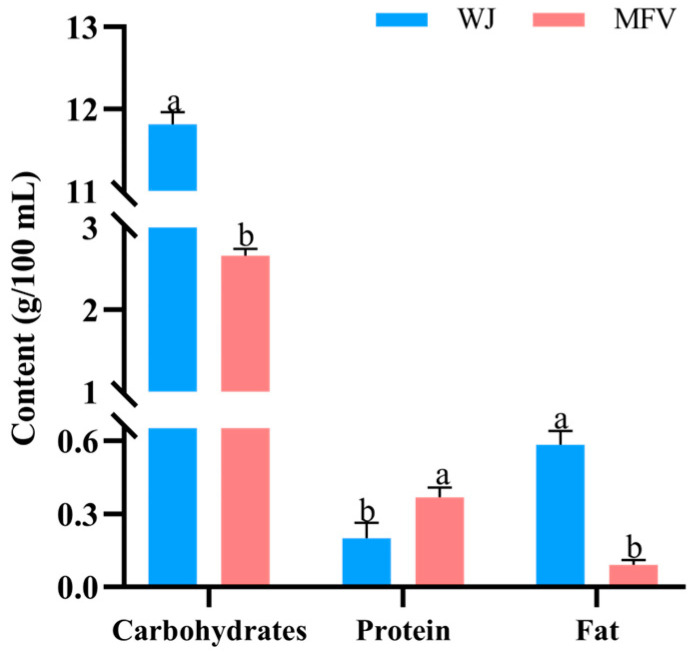
Nutritional components in mixed-culture fermented wolfberry vinegar (MFV). WJ: wolfberry juice. a, b: different lowercase letters present statistically significant differences (*p* < 0.05).

The nutritional components in WJ and MFV are shown in Figure 1. The content of carbohydrate was lower in MFV (2.66 ± 0.08 g/100 mL) than in WJ (11.65 ± 0.06 g/100 mL), which was due to the massive reproduction of yeast and lactic acid bacteria that consume carbohydrates during alcohol fermentation [22]. The protein and fat content were 0.36 ± 0.04 g/100 mL and 0.09 ± 0.02 g/100 mL, respectively. The protein content in MFV was significantly increased, while the fat content was significantly decreased, compared with that in WJ (*p* < 0.05). Guan et al. [23] reported that the fat content in traditional grain vinegar was 0.23 g/100 g, which is higher than the fat content in MFV. Tian et al. [2] reported that the protein and fat content of wolfberry vinegar fermented by yeast and acetic acid bacteria were 0.27 ± 0.01 g/100 mL and 0.12 ± 0.02 g/100 mL, respectively. These data indicate that MFV is a healthy product with low fat and carbohydrate levels.

### 3.2. Bioactive Ingredients and Antioxidant Activities in Wolfberry Juice and Mixed-Culture Fermented Wolfberry Vinegar

**Figure 2:**
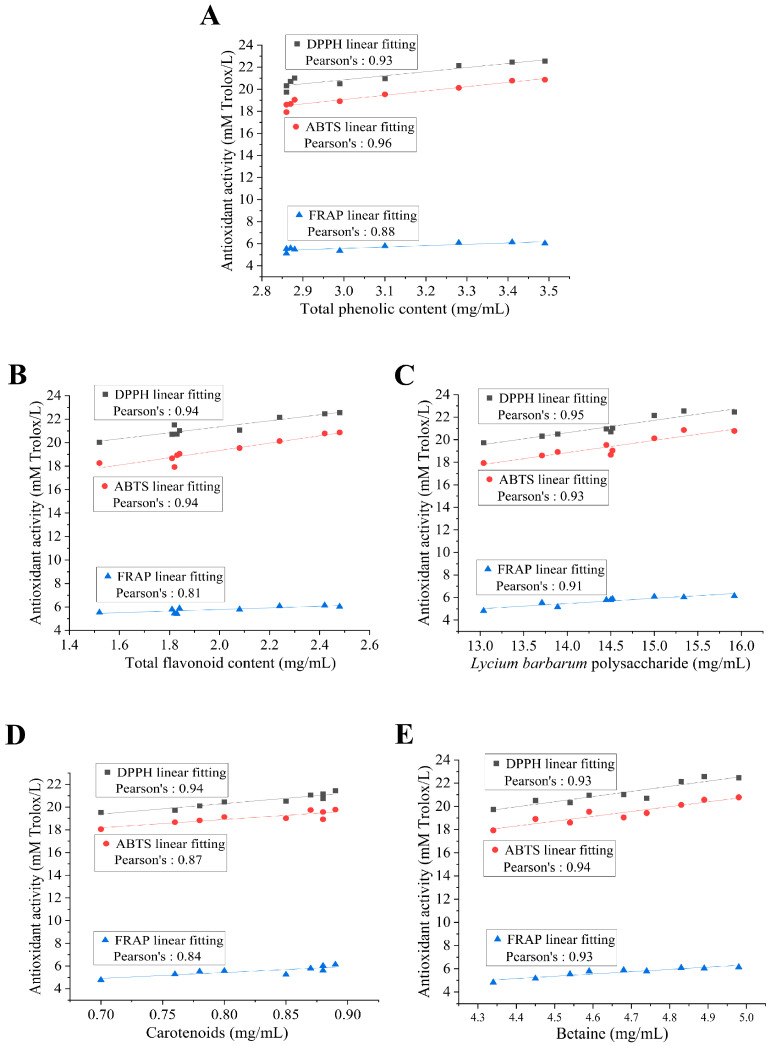
Correlation analysis of active ingredients and antioxidant activities in MFV. (**A**) Total phenolic content, (**B**) total flavonoid content, (**C**) *Lycium barbarum* polysaccharide (LBP), (**D**) carotenoids, and (**E**) betaine. DPPH: 1,1-diphenyl-2-picrylhydrazyl; ABTS: 2,2′-azino-bis(3-ethylbenzothiazoline)-6-sulfonic acid; FRAP: ferric ion reducing antioxidant power.

As shown in Table 1, the TPC and TFC in MFV were significantly raised compared with those in WJ (2.99 ± 0.12 mg GAE/mL and 1.81 ± 0.11 mg RE/mL, respectively (*p* < 0.05)). Wang et al. reported that the TPC in pineapple vinegar was 1.75 mg GAE/mL [24]. Another research study showed that the TPC (1.85 ± 0.07 mg GAE/mL) and TFC (0.44 ± 0.02 mg RE/mL) in citrus vinegar were higher than those in citrus juice [25]. These values were lower than those in MFV. The higher TPC and TFC of MFV compared with those of WJ may be attributed to the ability of lactic acid bacteria to produce more volatile phenols. LBP, as the main active ingredient, is used to evaluate the quality of wolfberry. The LBP content in MFV was 15.89 ± 0.12 mg/mL, which was higher than that in WJ (7.79 ± 0.38 mg/mL) (*p* < 0.05). The bright red color of wolfberry is also attributed to high levels of carotenoids. The carotenoid and betaine content were significantly increased compared with those in WJ (*p* < 0.05). Several studies reported that the betaine content of wolfberry vinegar and grain vinegar was 2.88 mg/mL and 0.59 mg/mL, respectively, which was lower than that of MFV in our study [21,26]. The antioxidant activities of WJ and MFV were detected by DPPH, ABTS, and FRAP. Compared with WJ, the DPPH, ABTS, and FRAP of MFV were increased by 66.54%, 68.82%, and 42.31%, respectively (*p* < 0.05). Moreover, correlation analysis was implemented to analyze bioactive ingredients and antioxidant activity (Figure 2). The correlation coefficients of active ingredients and antioxidant activity were both greater than 80. The correlation coefficient of TPC and ABTS was the highest (0.96), followed by LBP and DPPH (0.95). Therefore, the above results suggested that the TPC, TFC, LBP and betaine content, and antioxidant activities were increased after mixed-culture fermentation, which suggests that MFV is a beneficial health food with antioxidant properties. MFV can improve the value of products and meet consumer demand.

### 3.3. The Effect of Mixed-Culture Fermented Wolfberry Vinegar on Alcoholic Liver Disease in Mice

**Figure 3:**
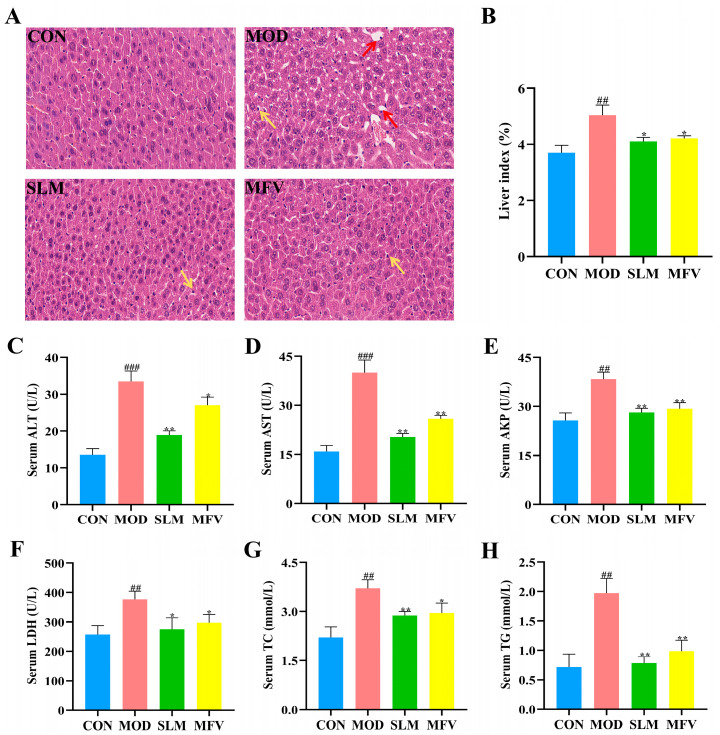
MFV alleviated the liver injury induced by alcohol in mice. (**A**) Effect of MFV on hepatic histopathological damage in alcohol-treated mice (H&E, 40×); red arrow indicates vacuolation, and yellow arrow indicates inflammatory cell. (**B**–**H**) Effect of MFV on serum alanine aminotransferase (ALT), aspartate aminotransferase (AST), alkaline phosphatase (AKP), lactate dehydrogenase (LDH), triglyceride (TG), and total cholesterol (TC) levels in alcohol-treated mice. Data are expressed as the mean ± SD (n = 8). ^##^ *p* < 0.01, ^###^ *p* < 0.001 vs. control group (CON), * *p* < 0.05, ** *p* < 0.01 vs. model group (MOD). SLM: silymarin group; MFV: mixed-culture fermented wolfberry vinegar group.

Excessive alcohol consumption leads to liver injury, including hepatic inflammation, apoptosis, and necrosis. As shown in Figure 3A, the liver cells of MOD showed structural disorders, vacuolation, and inflammatory cell infiltration in contrast to CON. However, the morphology of the liver cells in the MFV group and SLM group were similar to that in the control group: vacuolation and inflammatory infiltration were obviously improved in the ALD mice. In Figure 3B, the liver indexes were notably increased in MOD compared to those in CON (*p* < 0.05). However, pretreatment with SLM and MFV resulted in significant declines in liver indexes in alcohol-treated mice.

It is well known that alanine aminotransferase (ALT) and aspartate aminotransferase (AST) are important indicators of liver damage. As shown in Figure 3C,D, the content of ALT and AST in serum was evidently increased in MOD compared to that in CON (*p* < 0.05), indicating that alcohol consumption contributed to the hepatic damage. Pretreatment with MFV and SLM significantly decreased the serum levels of ALT and AST. The results showed that the activities of AKP and LDH in the MFV and SLM groups were significantly decreased in mice treated with alcohol (Figure 3E,F). Guo et al. studied the effects of goji powder on acute-induced hepatic injury in mice [27]. It was found that AST and ALT levels in MOD were obviously increased compared with those in CON, whereas these levels were significantly diminished in mice treated with goji. Another study reported that LBP (1% of feed weight) reduced the levels of AKP and LDH in mice treated with CCl4, which alleviated liver damage [28].

As illustrated in Figure 3G,H, the serum TC and TG levels were dramatically increased in MOD compared to those in CON, whereas MFV significantly inhibited the elevation of TC and TG levels (*p* < 0.05), indicating that MFV effectively facilitated lipid metabolism. These levels in the SLM group were similar to those in the MFV group. Yan et al. previously found that wolfberry (500 mg/kg·bw) significantly decreased TC levels in rats suffering from chronic alcoholic liver injury [29]. In conclusion, these results imply that MFV can effectively improve liver function in alcohol-treated mice.

### 3.4. The Effect of Mixed-Culture Fermented Wolfberry Vinegar on Alcohol Metabolism Enzymes in Mice with Alcoholic Liver Disease

**Figure 4:**
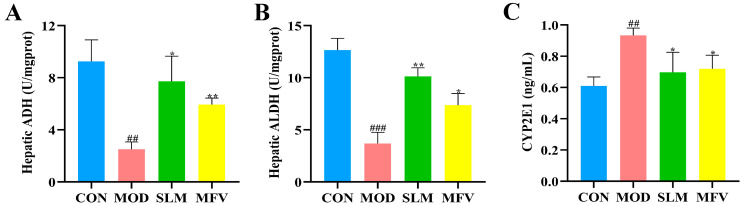
Effect of MFV on hepatic enzyme activities of (**A**) alcohol dehydrogenase (ADH), (**B**) acetaldehyde dehydrogenase (ALDH), and (**C**) cytochrome P450 (CYP2E1) in alcohol-treated mice. Data are expressed as the mean ± SD (n = 8). ^##^ *p* < 0.01, ^###^ *p* < 0.001 vs. control group (CON), * *p* < 0.05, and ** *p* < 0.01 vs. model group (MOD). SLM: silymarin group; MFV: mixed-culture fermented wolfberry vinegar group.

Alcohol is mainly metabolized through alcohol metabolism enzymes in the liver. As shown in Figure 4A,B, the activities of ADH and ALDH in MOD were significantly reduced compared with those in CON (*p* < 0.05), resulting in lower rates of oxidative metabolism of alcohol and increasing alcohol-related toxic effects in the liver. However, the levels of ADH and ALDH in the MFV group were remarkably increased compared with those in MOD. The results suggested that MFV accelerated alcohol metabolism in the liver by upregulating alcohol metabolism enzymes. As shown in Figure 4C, the MOD’s hepatic CYP2E1 protein levels were noticeably higher compared to those of the CON. Treatment with SLM and MFV noticeably downregulated the expression of CYP2E1 in alcohol-treated mice (*p* < 0.05). Lee et al. [30] found that persimmon vinegar significantly suppressed the expression of CYP2E1, and notably alleviated oxidative liver injury in rats. Taken together, these results suggest that MFV reverses alcohol metabolism enzymes and reduces CYP2E1 activity, which further alleviates alcohol-induced liver injury.

### 3.5. The Effect of Mixed-Culture Fermented Wolfberry Vinegar on Liver Oxidative Stress in Mice with Alcoholic Liver Disease

**Figure 5:**
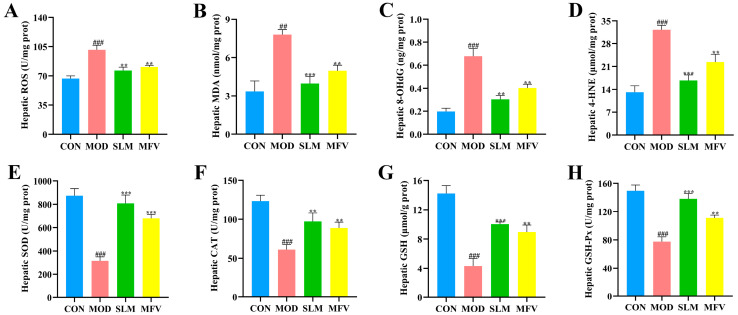
Effects of MFV on oxidative stress and levels of of hepatic (**A**) reactive oxygen species (ROS), (**B**) malondialdehyde (MDA), (**C**) 8-hydroxy-2′-deoxyguanosine (8-OHdG), (**D**) 4-hydroxynonenal (4-HNE), (**E**) superoxide dismutase (SOD), (**F**) catalase (CAT), (**G**) glutathione (GSH), and (**H**) glutathione peroxidase (GSH-Px) in alcohol-treated mice. Data are expressed as the mean ± SD (n = 8). ^##^ *p* < 0.01, ^###^ *p* < 0.001vs. control group (CON), ** *p* < 0.01, *** *p* < 0.001 vs. model group (MOD). SLM: silymarin group; MFV: mixed-culture fermented wolfberry vinegar group.

Excessive ROS are produced in the process of alcohol metabolism, which leads to oxidative stress [14]. Oxidative stress caused by increased pro-oxidant levels and decreased antioxidant levels is a major driver of alcohol-induced liver damage [31]. As shown in Figure 5A–D, the levels of ROS, 8-OHDG, 4-HNE, and MDA in the model group were dramatically increased compared with those in CON, indicating that excessive alcohol consumption led to excessive oxidative products in the liver. In contrast, these oxidative indicators were significantly decreased in the MFV group (*p* < 0.05). These results indicate that MFV could effectively inhibit elevated levels of oxidation in alcohol-treated mice.

SOD, CAT, GSH, and GSH-Px can lower oxidative stress levels by eliminating excess reactive oxygen species and free radicals [32]. In Figure 5E–H, the activities of SOD, CAT, GSH-Px, and GSH in the model group were dramatically lower than those in the control group, indicating that alcohol exposure led to the depletion of endogenous antioxidant enzymes and GSH content. Nevertheless, MFV significantly alleviated the decrease in SOD, CAT, GSH-Px, and GSH (*p* < 0.05). Taken together, our data suggest that MFV pretreatment regulates the equilibrium between pro-oxidant and antioxidant levels, which further prevents alcohol-induced oxidative injury in the liver.

### 3.6. Effect of Mixed-Culture Fermented Wolfberry Vinegar on Hepatic Inflammatory Indicators in Mice with Alcoholic Liver Disease

**Figure 6:**
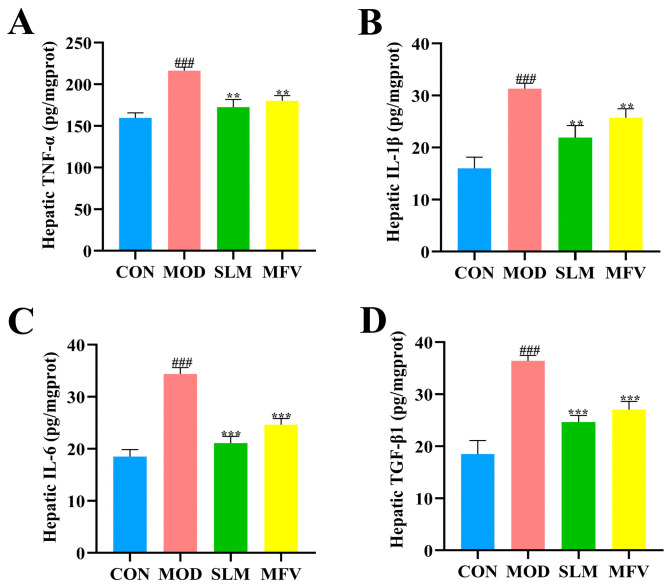
Effects of MFV on liver inflammation and levels of (**A**) tumor necrosis factor-α (TNF-α), (**B**) interleukin-1β (IL-1β), (**C**) interleukin-6 (IL-6), and (**D**) transforming growth factor-β1 (TGF-β1) in alcohol-treated mice. Data are expressed as the mean ± SD (n = 8). ^###^ *p* < 0.001 vs. control group (CON), ** *p* < 0.01, and *** *p* < 0.001 vs. model group (MOD). SLM: silymarin group; MFV: mixed-culture fermented wolfberry vinegar group.

As shown in Figure 6A–D, the TNF-α, IL-1β, IL-6, and TGF-β1 levels in the model group were noticeably higher than those in CON (*p* < 0.05), suggesting that alcohol exposure led to an elevation in cytokine levels in hepatocytes. These levels were considerably decreased in the SLM and MFV groups, which contrasted with those in model group (*p* < 0.05). Xia et al. [33] indicated that *Lycium ruthenicum Murray* (375 mg LRM/kg b.w.) reduced alcohol-induced inflammatory responses by reducing TNF-α, IL-1β, and IL-6 concentrations in ALD mice, which is similar to our results. These results suggest that MFV might protect against alcohol-induced liver injury by inhibiting the release of proinflammatory cytokines.

### 3.7. Effect of Mixed-Culture Fermented Wolfberry Vinegar on the Alcohol-Induced PI3K-AKT-NF-κB Pathway In Vivo

**Figure 7:**
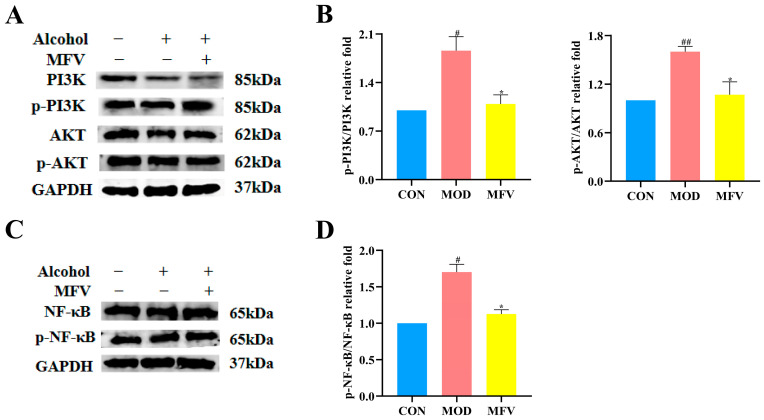
Effect of MFV on PI3K-AKT-NF-κB protein expression. (**A**) The expression of PI3K, p-PI3K, AKT, and p-AKT proteins by western blotting. (**B**) Quantified levels of hepatic p-PI3K/PI3K and p-AKT/AKT expression. (**C**) The expression of NF-κB and p-NF-κB proteins by western blotting. (**D**) Quantified levels of hepatic p-NF-κB/NF-κB expression. ^#^ *p* < 0.05, ^##^ *p* < 0.01 vs. control group (CON), and * *p* < 0.05 vs. model group (MOD). SLM: silymarin group; MFV: mixed-culture fermented wolfberry vinegar group.

The PI3K/AKT signaling pathway is suppressed to inhibit the activation of the NF-κB signaling pathway, which has become a potential liver-protective strategy. As shown in Figure 7, a western blot analysis of liver tissue revealed that the expression levels of the inflammation-related proteins p-PI3K, PI3K, p-AKT, AKT, p-NF-κB, and NF-κB were significantly higher in the model group than those in the control group, while the levels of p-PI3K/PI3K, p-AKT/AKT, and p-NF-κB/NF-κB were significantly lower than those in the MFV group (*p* < 0.05), indicating that MFV alleviated alcohol-induced liver injury. Ma et al. [34] reported that 40 mg/kg of total flavonoids from *Carthamus tinctorius* L. leaves reduced the levels of the inflammatory factors TNF-α, IL-6, and IL-1β, downregulated the expression levels of PI3K/AKT, inhibited NF-κB p65, and had a liver-protective effect in mice with CCl_4_-induced liver injury. These data imply that MFV protects against ALD by inhibiting the activation of the PI3K-AKT-NF-κB pathway in vivo.

## 4. Conclusions

Consequently, the present study demonstrated that MFV is a healthy product with low fat and carbohydrate levels. MFV had higher levels of bioactive ingredients, including phenolics, flavonoids, LBP, and betaine, after mixed-culture fermentation, which had a high correlation with antioxidant activities. Moreover, MFV alleviated alcohol-induced liver injury by improving histopathological changes, the expression of alcohol metabolizing enzymes, and antioxidant indexes in alcohol-treated mice. Meanwhile, MFV reduced liver biochemical indicators, the expression of CYP2E1, and oxidative markers. Moreover, MFV reduced the inflammatory response by inhibiting the PI3K/Akt/NF-κB signaling pathway. This study provides compelling evidence that MFV can serve as a natural hepatoprotective functional food. Future research studies should explore MFV in human trials and its potential for industrial scalability.

## Figures and Tables

**Table 1:** Content of active ingredients and antioxidant activities in wolfberry juice (WJ) and mixed-culture fermented wolfberry vinegar (MFV).

	Indexes	Content	
		WJ	MFV
Active ingredients	TPC (mg GAE/mL)	1.38 ± 0.03 b	2.99 ± 0.12 a
	TFC (mg RE/mL)	1.02 ± 0.06 b	1.81 ± 0.11 a
	LBP (mg/mL)	7.79 ± 0.38 b	15.89 ± 0.12 a
	Carotenoids (mg/mL)	0.87 ± 0.06 a	0.86 ± 0.05 a
	Betaine (mg/mL)	3.01 ± 0.06 b	4.74 ± 0.14 a
Antioxidant activities	DPPH (mM Trolox/L)	6.95 ± 0.32 b	20.84 ± 0.13 a
	ABTS (mM Trolox/L)	5.82 ± 0.49 b	18.67 ± 0.07 a
	FRAP (mM Trolox/L)	3.34 ± 0.24 b	5.79 ± 0.26 a

Data are presented as mean ± standard deviation of three sets of replicate experiments. a, b: values in the same row with different letters are significantly different at *p* < 0.05. TPC: total phenolic content; TFC: total flavonoid content; LBP: *Lycium barbarum* polysaccharide; DPPH: 1,1-diphenyl-2-picrylhydrazyl; ABTS: 2,2′-azino-bis(3-ethylbenzothiazoline)-6-sulfonic acid; FRAP: ferric ion reducing antioxidant power.

## Data Availability

Data are contained within the article.
